# Green fluorescent nanomaterials for rapid detection of chromium and iron ions: wool keratin-based carbon quantum dots†

**DOI:** 10.1039/d2ra00529h

**Published:** 2022-03-11

**Authors:** Yuanyuan Song, Na Qi, Kang Li, Di Cheng, Dong Wang, Ying Li

**Affiliations:** Key Lab. of Colloid and Interface Chemistry of State Education Ministry, Shandong University Jinan 250100 China yingli@sdu.edu.cn +86 531 88364464 +86 531 88362078

## Abstract

Heavy metal ions produced by industrial activity have been a serious environmental problem, and their detection is critical for treatment of the heavy metal pollution. Among the variable heavy metal detection methods, fluorescent indicator methods have attracted wide attention due to the advantage of the convenience and nondestructive detection process. Carbon quantum dots have great application in this respect. In this study, nitrogen and sulfur doped fluorescent carbon quantum dots (N,S-CDs) were prepared based on wool keratin using a hydrothermal method, of which the morphology, chemical composition and optical properties were characterized. The prepared N,S-CDs were spherical nanoparticles with a diameter of 2–6 nm, showing wide fluorescence excitation and emission wavelength range and considerable quantum yield, and have sensitive response to Cr^6+^ and Fe^3+^. This study reveals a novel cost-effective and convenient synthetic route of green carbon quantum dots through naturally sourced materials, demonstrates the application potential of the wool keratin-based N,S-CDs in rapid detection of heavy metal, and opens up a new path for functional utilization of waste wool keratin.

## Abstract 2

Synthesis of carbon quantum dots from wool keratin and their potential in detecting chromium and iron ions.

## Graphical abstract

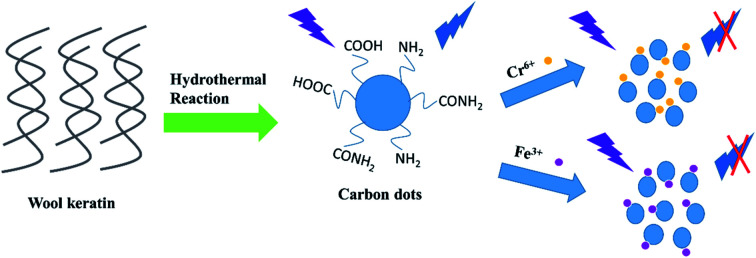

## 1. Introduction

The global industrial revolution and rapid urbanization have escalated rapidly and resulted in the generation of huge quantities of wastewater all over the world.^1^ In various industrial processes, such as electroplating, leather tanning, pigment production,^2–5^*etc.*, diverse heavy metal ions are discharged with the produced wastewater, leading to the risk of causing irreversible damage to the environment and human body.^2,6–8^ The detection and treatment of heavy metal ions in water has become a serious issue that needs to be solved urgently. The common detection methods for heavy metal ions usually relied on large instruments such as atomic absorption spectrometry (AAS) and inductively coupled plasma-mass spectrometry (ICP-MS),^9,10^ which are expensive and have complicated in sample pre-treatment.

In recent years, fluorescent indicators have been developed for the detection of heavy metal ions, which could be convenient and rapid.^11^ Among various fluorescent materials, carbon quantum dots (CDs) gained lots of attention due to the environmental-friendly composition.^12–14^ Several synthesis methods of carbon quantum dots have been developed, including laser ablation, arc discharge, electrochemical method, microwave method, hydrothermal method, *etc.*,^15^ among which hydrothermal method is attracting attention for the lower energy consumption and convenient operation. Small molecules such as citric acid,^16^ glucose^17^ and amino acid^18–21^ have been used as precursors for the synthesis of carbon dots by hydrothermal method. And some natural sourced polymers, such as milk, juice, cyanobacteria, grass.^22–26^

Keratin was a kind of natural macromolecule which could be extracted from many animal feathers, hairs and horns, *etc.*^27^ Wool keratin is an α-keratin and the most important constituent amino acid is cysteine, which contains sulfur, so wool keratin contains sulfur.^28^ The existence of abundant N and S containing groups in keratin molecules give it high potential in synthesizing CDs with high quantum yield, since N,S doping at CDs have been proved to be very effective in regulating the energy band gap and electron density of CDs.^29,30^ Ai *et al.* synthesized carbon naonospheres using hair, and the fluorescence quantum yield is desirable.^31^ Synthesizing green CDs from keratin could be sustainable and environmental-friendly way due to the abundant reserves, while the synthetic pathway, the fluorescent properties and application performance of wool keratin based-CDs need to be extensively explored.

In this study, bio-friendly N,S-doped CDs with high quantum yield were synthesized based on wool keratin using hydrothermal method under 200 °C. The chemical composition and fluorescence properties of the prepared CDs were investigated. The prepared CDs showed characteristic fluorescence quenching effect in the presence of Cr^6+^ or Fe^3+^, which made it a potential selective detection indicator for Cr^6+^. Iron ions sometimes interfere with the detection of chromium ions, but the interference of iron ions with it can be eliminated by adding acid. Due to the sensitivity of the prepared carbon dots for chromium ion detection, this method shows the application of chromium ion detection in electroplating wastewater. Chromium ions are the main pollutant in electroplating wastewater. The mechanisms were discussed. On the one hand, this study attempts to propose an effective way to synthesize green CDs and explore innovative fluorescent nanomaterials that can be conveniently used for heavy metal ion detection, on the other hand, it aims to explore a utilization approach of keratin derived from the waste wool, which could be also meaningful.

## 2. Experimental section

### 2.1 Materials

Wool keratin was purchased from TCI Shanghai Chemical Industry Development Co., Ltd. (Total nitrogen: 14.0 to 16.0%, the content of sulfur element is 1.42%, as shown as Fig. S1†). Standard stock (0.1 mol L^−1^) solution of Cr^6+^, Fe^3+^ ions was respectively prepared with K_2_Cr_2_O_7_, FeCl_3_ in ultrapure water. Different concentrations of Cr^6+^, Fe^3+^ ions were obtained by diluting standard stock solutions. Other stock solutions (0.5 mM) of metal cations were prepared in deionized water from the respective salts of CdCl_2_, Pb(NO_3_)_2_, MnCl_2_·2H_2_O, NiCl_2_, CuCl_2_, CaCl_2_, MgCl_2_, BaCl_2_, AlCl_3_, FeCl_2_, Cr(NO_3_)_3_. And all aqueous solutions were prepared with distilled water produced by a Milli-Q system (Millipore, USA, 18.25 MΩ cm). Chitosan with a degree of deacetylation (DA) of more than 95% and *N*,*N*′-methylenebisacrylamide were purchased from Shanghai Macklin Biochemical Co., Ltd. Acrylic acid was purchased from Tianjin Damao Chemical Reagent Factory.

### 2.2 Synthesis of N,S-CDs

Initially, a mixture of wool keratin (0.2 g) and distilled water (10 mL) was stirred for 30 minutes, sonicated (100 W) for 5 minutes, and then transferred to a 25 mL Teflon-lined pressure vessel and heated at 180 °C or 200 °C for 8–12 h. After the reaction was completed, the reaction solution was cooled to room temperature, centrifuged at 8500 rpm for 10 minutes, and filtered through a 0.22 μm microporous membrane. The prepared N,S-CDs solution was stored at 4 °C and used in the further determination.

### 2.3 Characterization

The morphology and size of N,S-CDs were analyzed by transmission electron microscope (HT-7700, Japan) operated at 100 kV. Fourier transform infrared (FT-IR) spectra were obtained on a Thermo Scientific Nicolet iS5 IR spectrometer (USA) ranging from 400 to 4000 cm^−1^ using attenuated total reflection (ATR) accessory. XPS analysis was measured by Thermo Scientific ESCALAB Xi^+^ X-ray photoelectron spectrometer (USA) using Al Kα as the excitation source (1486.6 eV).

### 2.4 Optical property determination

Fluorescence spectroscopy was performed with a Horiba FluoroMax-4 spectrofluorometer (Japan) at different excitation wavelength ranging from 365 to 520 nm and 270, 323 nm. UV-vis absorption spectra were obtained using a UV-1800 Shimadzu UV spectrophotometer (Japan). Fluorescence lifetime was measured by an Edinburgh FLS920 Fluorescence Spectrometer (England) with excitation and emission wavelengths of 365 and 434 nm respectively.

### 2.5 Quantum yield calculations

The absolute quantum yield of the sample was determined by FLS920 Fluorescence Spectrometer (Edinburgh, England) using an integrating sphere. The excitation wavelength was set to 365 nm and the emission wavelength range was 355–600 nm. The excitation scattering peak and emission peak of the reference and sample were tested, and the blank test was performed using pure water.

The quantum yield (QY) of the sample is automatically calculated using the following equation:1
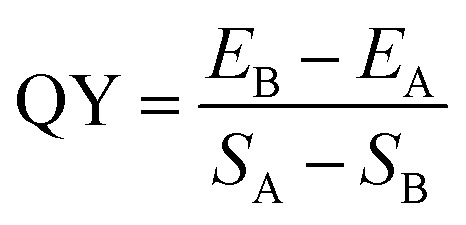
*E*_A_, *E*_B_ are the integrated areas of the emission peaks for the reference and sample, respectively. *S*_A_, *S*_B_ are the integrated areas of the scattering peaks for the reference and sample, respectively.

### 2.6 The influence of different metal ions on the fluorescence properties of N,S-CDs

The solutions containing different metal ions were diluted to 5 mM. 2 ml 5 mM metal ion solution was mixed with 2 ml N,S-CDs solution. The fluorescence emission of the mixture was observed under 365 nm UV light irradiation, and the relative fluorescence intensity under 365 nm UV light irradiation was recorded with a fluorescence spectrometer (Horiba FluoroMax-4).

### 2.7 Quenching effect of Cr^6+^ and Fe^3+^ on the fluorescence of N,S-CDs

Different concentrations of K_2_Cr_2_O_7_ and FeCl_3_ solutions (0.5, 5, 10, 50, 250, 500, 1000, 2500, 5000 μM) were prepared. The Cr^6+^ or Fe^3+^ solution was mixed with N,S-CDs solution in the mass ratio of 1 : 1, and the mixture was observed for fluorescence emission under UV light irradiation, and the relative fluorescence intensity of the mixture was tested by fluorescence spectrometer (Horiba FluoroMax-4).

### 2.8 Synthesis of fluorescent CDs-hydrogel

0.1 g chitosan was dissolved in 6 ml 0.1 M acetic acid solution and stirred until completely dissolved, then 400 μL N,S-CDs solution was added to the above solution. 5 wt% ammonium persulfate solution was added into the chitosan solution under stirring. After reacted for 10 min, 2 ml acrylic acid and 0.2 g *N*,*N*′-methylenebisacrylamide was added into the above solution one after another and kept stirred until dissolved. Then the dissolved solution was heated at 60 °C for 3 h. Finally, the fluorescent CDs-hydrogel was obtained.

## 3. Results and discussion

### 3.1 Hydrothermal synthesis of wool keratin-based carbon quantum dots

The environmentally friendly hydrothermal method was used to synthesize carbon dots, the reaction temperature was controlled no more than 200 °C to ensure low energy consumption, and natural sourced wool keratin was used as the precursor, through which the green synthesis of carbon quantum dots was realized.

Wool keratin, which is insoluble under room temperature, can be degraded into small amino acid molecules under hydrothermal conditions. The small amino acid molecules undergo a dehydration condensation reaction with each other, and further intermolecular polymerization, and then carbonization to form carbon nanomaterials.^31^

**Fig. 1:**
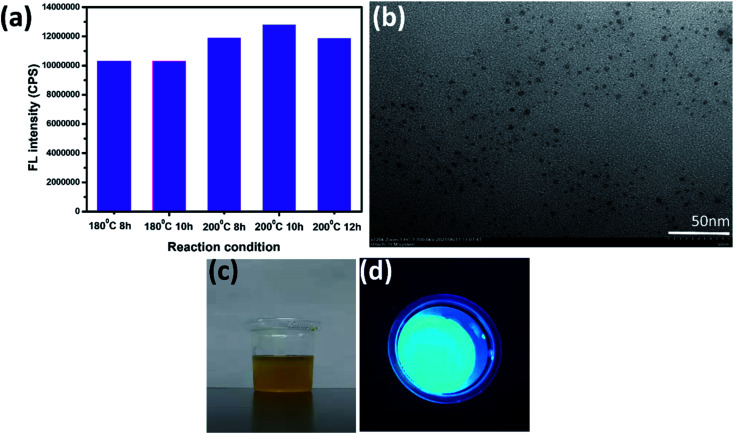
(a) The fluorescence intensity of the wool keratin-based CDs obtained by hydrothermal reaction under different conditions (different reaction conditions: 180 °C 8 h, 180 °C 10 h, 200 °C 8 h, 200 °C 10 h, 200 °C 12 h). (b) TEM image of the prepared wool keratin based-CDs. (c) The pictures of the aqueous wool keratin-CDs solution taken under daylight and (d) under UV light.

The effect of temperature and reaction time on the fluorescence intensity of the hydrothermally synthesized carbon dots has been investigated (Fig. 1(a)), through which the optimal reaction conditions for the synthesis of carbon dots from wool keratin were determined to be heated at 200 °C for 10 h. The morphology of the as-prepared carbon dots was characterized by TEM, as shown in Fig. 1(b), the carbon dots are spherical nanoparticles with a size of about 2–6 nm. The solution of the carbon dots exhibited pale yellow color under daylight and bright blue color under excitation of 365 nm UV light (Fig. 1(c) and (d)).

### 3.2 Chemical composition analyzation of the prepared wool keratin based-CDs

**Fig. 2:**
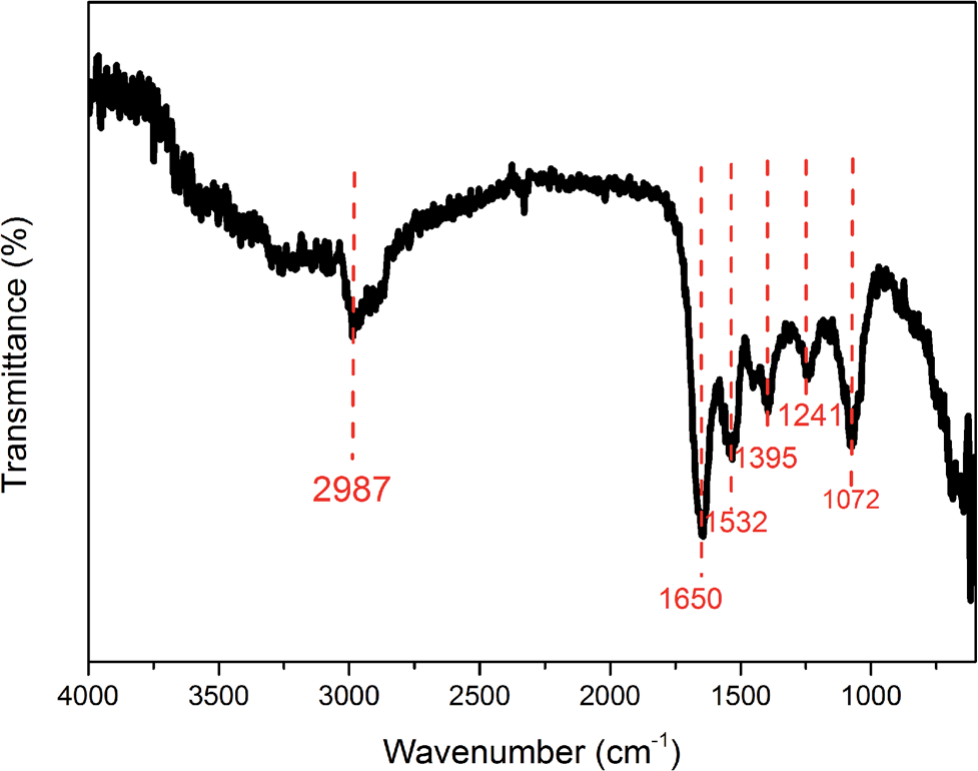
FT-IR spectra of wool keratin-CDs.

The FT-IR measurements are carried using ATR accessory to analyze the surface chemical composition and structural information of the prepared carbon dots (Fig. 2). The characteristic absorption band of C=O around 1650 cm^−1^, the bending vibration band of N–H at 1532 cm^−1^, and the stretching vibration band of C–N at 1241 cm^−1^ indicate that there are amide groups, carboxylic acid groups and other oxygen-containing functional groups on the surface of the N,S-CDs. It could be proved that there are considerable amount of nitrogen element existing on the surface of the prepared carbon dots. Furthermore, the peak at 2987 cm^−1^ is attributed to the stretching vibration of C–H, suggesting the presence of alkyl groups. And the peak at 1072 cm^−1^ is associated with the stretching vibration of S–O, suggesting sulfur element is also doped on the surface of the prepared carbon dots.

**Fig. 3:**
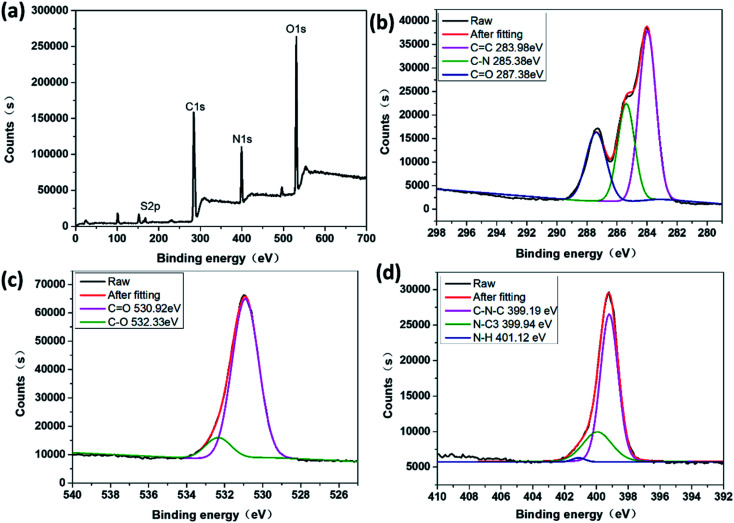
XPS spectra of the prepared wool keratin based-CDs. (a) Survey spectrum. (b) C 1s spectrum. (c) O 1s spectrum. (d) N 1s spectrum.

The surface states of the prepared carbon dots were charcaterized by X-ray photoelectron spectroscopy. The XPS spectrum (Fig. 3(a)) of the prepared carbon dots shows typical peaks of C_1s_, N_1s_, O_1s_ and S_2p_. The N content is as high as 14.05%, which is much higher than that reported in the past.^31^ The C_1s_ spectrum (Fig. 3(b)) exhibits three peaks at 283.98, 285.38 and 287.38 eV, which are attributed to C=C, C–N and C=O, respectively, which are in agreement with the FT-IR results. The spectrum of O_1s_ (Fig. 3(c)) shows two peaks at 530.92 and 532.33 eV, which are attributed to C=O and C–O groups, respectively. The N 1s spectrum (Fig. 3(d)) reveals three peaks at 399.19, 399.94 and 401.12 eV, which are attributed to C–N–C, N–C_3_ and N–H. The above results confirm the existence of C=C, C–O, C–N, N–H, COOH bonds and clearly show that the prepared CDs are functionalized with amide, hydroxyl, amino, carbonyl and carboxylic acid groups. In most situations, the PL color of CDs is relative to the surface groups rather than the size. After surface modification or passivation, the QY can be dramatically increased. The enhanced PL properties are attributed to the strongly PL centers on the surface, the synergy between the carbon core and the chemical groups, or solely to the presence of fluorophores.^32^

### 3.3 Optical property characterization of N,S-CDs

**Fig. 4:**
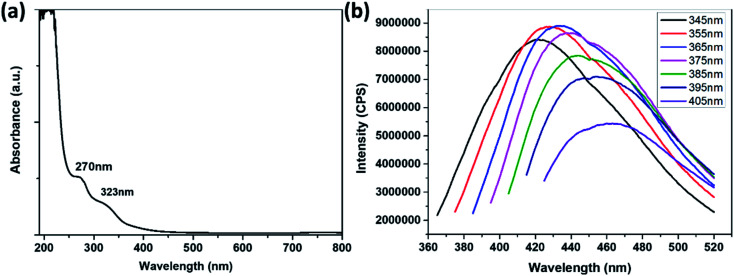
(a)UV-vis absorption spectrum of N,S-CDs. (b)PL emission spectra of N,S-CDs at different excitation wavelengths.

The UV absorption spectrum and the PL emission spectra of the prepared N,S-CDs are shown in Fig. 4. The prepared N,S-CDs have the common optical absorption characteristic of carbon dots, that is, they have obvious optical absorption in the UV light region, with a tail extending to the visible range (Fig. 4(a)).^13^ The UV-visible absorption spectrum of N,S-CDs has two absorption peaks, and the peak at 270 nm is assigned to the π–π* transition of C=C on the surface of the carbon dots, and the peak at 323 nm is assigned to the n–π* transition of the C=O or other connected functional groups on the surface of the carbon dots.

The fluorescence emission spectra of the N,S-CDs at different excitation wavelengths were tested. It was found that the emission wavelength and fluorescence intensity varied with excitation wavelength (Fig. 4(b)), similar to those previously prepared in other literature.^33^ The fluorescence emission spectra of the N,S-CDs under excitation at 270 and 323 nm are shown in Fig. S4.† The maximum fluorescence emission wavelength of the prepared N,S-CDs is 434 nm, and the maximum fluorescence excitation wavelength is 365 nm, and the maximum quantum yield measured is 8%. With the increase of excitation wavelength, the emission wavelength was red-shifted, the relative fluorescence intensity at the maximum emission wavelength first increased and then decreased. The reason for the red shift is related to the excitation-dependent PL behavior of N,S-CDs, which was correspond to the properties of carbon quantum dots by previous publications.^34,35^ This excitation wavelength-dependent property may be caused by the selection of nanoparticles based on their size (quantum effect) or different emissive traps on the surface of N,S-CDs.^36^

### 3.4 The influence of different metal ions on the fluorescence property of the N,S-CDs

**Fig. 5:**
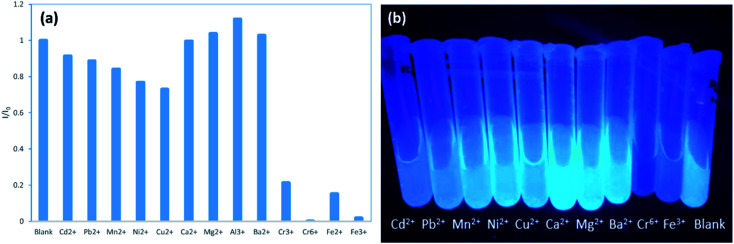
(a) Relative fluorescent intensity at *λ*_ex_ = 365 nm of aqueous N,S-CDs solution in the presence of 2.5 mM of various metal ions. (b) Photograph of the aqueous N,S-CDs solution containing 2.5 mM of various metal ions under UV light (excited at 365 nm) (various metal ions: Cd^2+^, Pb^2+^, Mn^2+^, Ni^2+^, Cu^2+^, Ca^2+^, Mg^2+^, Ba^2+^, Cr^6+^, Fe^3+^).

The variation of the PL intensity of N,S-CDs solution in the presence of various metal ions (including Cd^2+^, Pb^2+^, Mn^2+^, Ni^2+^, Cu^2+^, Ca^2+^, Mg^2+^, Ba^2+^, Al^3+^, Fe^2+^, Cr^3+^, Cr^6+^ and Fe^3+^) were tested under the same conditions (Fig. 5(a), (b), S3, S4 and S6†). It was found that both Cr^6+^ and Fe^3+^ could significantly quench the fluorescence of N,S-CDs. When the N,S-CDs were added to the Cr^6+^ solution of 2.5 mM, the fluorescence of the N,S-CDs solution was almost completely quenched. While when the N,S-CDs were added to the Fe^3+^ solution of 2.5 mM, the fluorescence intensity of N,S-CDs solution was reduced by 98.2%. The results indicated that the prepared N,S-CDs is sensitive to the presence of Cr^6+^ and Fe^3+^.

### 3.5 The capacity of N,S-CDs used as probe for Cr^6+^ and Fe^3+^ detection

**Fig. 6:**
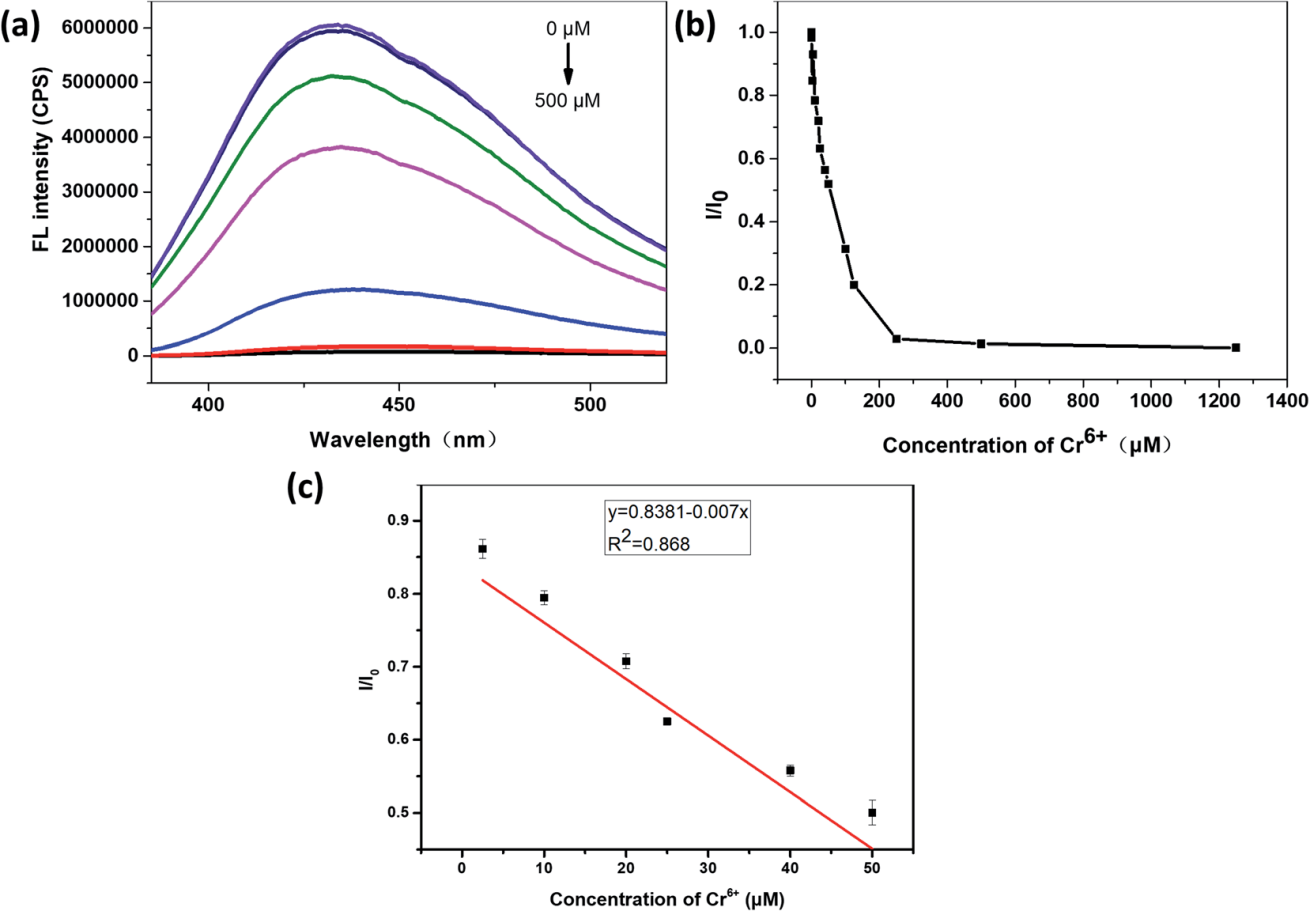
(a) Fluorescence emission spectra of the N,S-CDs in aqueous medium upon addition of various concentrations of Cr^6+^ (the concentration of Cr^6+^ solution from top to bottom is in the order of 0, 0.25, 2.5, 25, 125, 250, 500 μM), excitated at 365 nm. (b) The relationship between *I*/*I*_0_ and concentration of Cr^6+^ ion (the concentration of Cr^6+^ ion is from 0 to 1250 μM). (c) The linear region between *I*/*I*_0_ and concentrations of Cr^6+^ ions (the concentration of Cr^6+^ ion is from 2.5 to 50 μM).

It can be seen from Fig. 6(a) and (b) that the relative fluorescence intensity of N,S-CDs gradually decreases with increasing Cr^6+^ concentration. As it was shown in Fig. 6(c), when the concentration of Cr^6+^ was in the range of 2.5–50 μM, *I*/*I*_0_ (*I*_0_ and *I* are the PL intensities of N,S-CDs excited at 365 nm in the absence and presence of Cr^6+^ ion, respectively) has a linear relationship with the concentration of Cr^6+^. The linear equation for the detection of chromium ions by the prepared N,S-CDs is *I*/*I*_0_ = −0.007*C* (Cr^6+^) + 0.8381 (*R*^2^ = 0.868). The calculated limit of detection (LOD) is 14.16 nM (3*σ*/*k*). Test experiments were repeated three times.

**Fig. 7:**
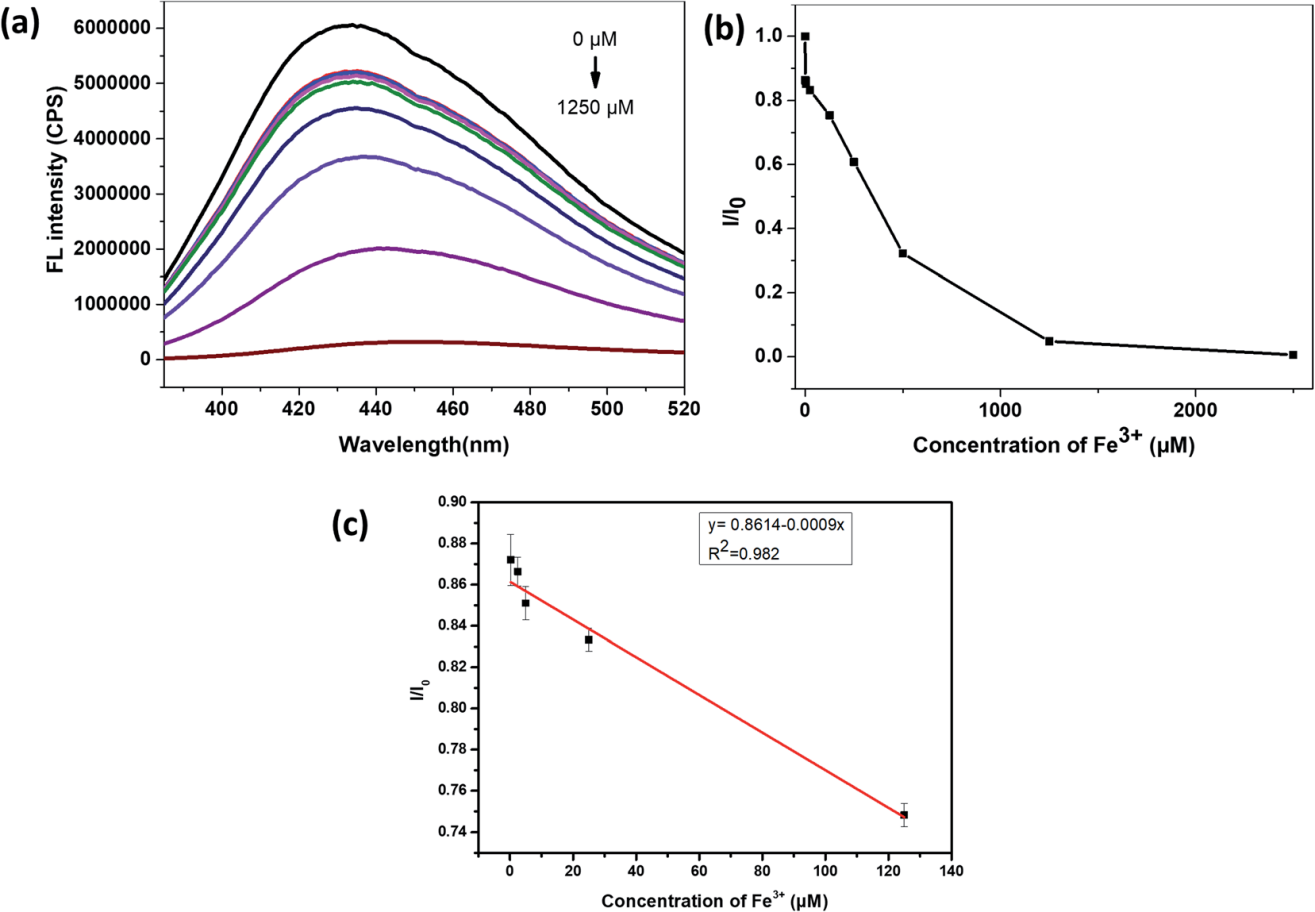
(a) Fluorescence emission spectra of the N,S-CDs in aqueous medium upon addition of various concentrations of Fe^3+^ (the concentration of Fe^3+^ solution from top to bottom is in the order of 0, 0.25, 2.5, 10, 25, 125, 250, 500, 1250 μM), excited at 365 nm. (b) The relationship between *I*/*I*_0_ and concentrations of Fe^3+^ ions (the concentration of Fe^3+^ ion is from 0 to 2500 μM). (c) The linear region between *I*/*I*_0_ and concentrations of Fe^3+^ ions (the concentration of Fe^3+^ ion is from 0.25 to 125 μM).

Similarly, it can be seen from Fig. 7(a) and (b) that, the relative fluorescence intensity of N,S-CDs gradually decreases with the increase of Fe^3+^ concentration, which also revealed that the prepared N,S-CDs is sensitive to the Fe^3+^ concentration. And when the concentration of Fe^3+^ was in the range of 0.25–125 μM, *I*/*I*_0_ (*I*_0_ and *I* are the PL intensities of N,S-CDs excited at 365 nm in the absence and presence of Fe^3+^ ion, respectively) also showed good linearity with the concentration of Fe^3+^ (Fig. 7(c)). The linear equation for the detection of iron ions by the prepared N,S-CDs is *I*/*I*_0_ = −0.0009*C* (Fe^3+^) + 0.8614 (*R*^2^ = 0.982). The calculated limit of detection (LOD) is 113 nM (3*σ*/*k*). Comparison of this method with other detection methods by carbon dots reported in the literature is presented in Table 1.

**Table 1:** Comparison of the proposed methods with previous methods employed for Cr^6+^ detection based on carbon quantum dots

Detection probes	Linear range	Detection limit	Ref.
Wool keratin based carbon dots	2.5–50 μM	0.014 μM	This work
Shrimp shell based carbon dots	0–70 μM	0.1 μM	37
Flax straw based carbon dots	0.5–80 μM	0.19 μM	38
Natural kelp based carbon dots	0.01–50 μM	0.52 μM	39
Dual emission carbon dots from *m*-aminophenol and oxalic acid	2–300 μM	0.4 μM	40

The application direction of this method is the detection of the wastewater generated in electroplating industry where chromium ions are the main pollutant. The electroplating industry produces a variety of pollutants, among which heavy metals include Cr, Ni, Cu and Zn. Chromium ion is one of the heavy metal ions with high toxicity, so timely detection of the presence of chromium ion in the water environment is necessary. For the above application purpose, a simulating electroplating wastewater solution including Cr^6+^, Ni^2+^, Cu^2+^ and Zn^2+^ ions, were used to test whether the fluorescence of N,S-CDs could be quenched by chromium ions under the interference with the other types of cations. The test results showed that in the presence of Ni^2+^, Cu^2+^ and Zn^2+^ ions, the fluorescence of the prepared carbon dots was still quenched with encountering Cr^6+^ ions (Fig. S5†), and the prepared carbon dots could still achieve the detection of Cr^6+^ ions.

### 3.6 The mechanism of the fluorescence quenching of N,S-CDs by Cr^6+^ and Fe^3+^

**Fig. 8:**
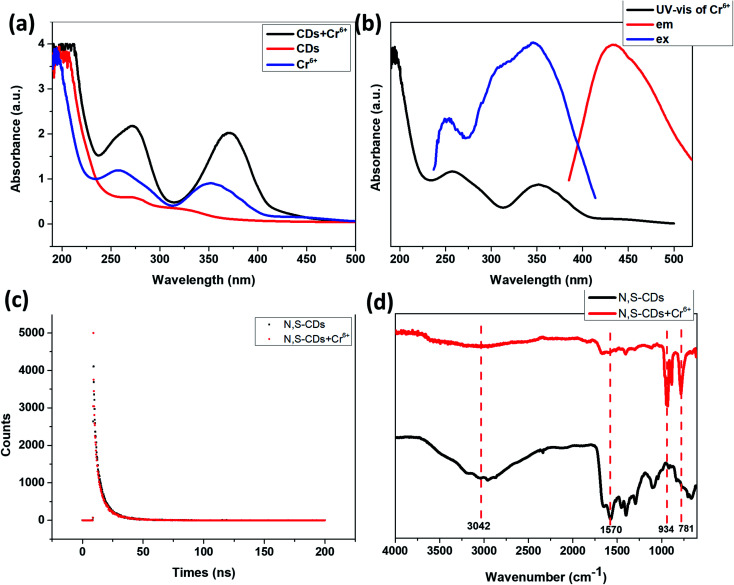
(a) UV-vis absorption spectra of Cr^6+^, N,S-CDs in the absence and presence of Cr^6+^. (b) UV-vis absorption spectra of Cr^6+^ and fluorescence excitation and emission spectra of N,S-CDs. (c) Lifetime test of N,S-CDs in the absence and presence of Cr^6+^ (excitation wavelength is at 365 nm, emission wavelength is at 434 nm). (d) FT-IR spectra of N,S-CDs in the absence and presence of Cr^6+^.

The UV-vis absorption spectra of N,S-CDs solution in the absence and presence of Cr^6+^ was shown in Fig. 8(a). It can be seen that the presence of Cr^6+^ could significantly change the absorption spectrum of the N,S-CDs. The UV-vis absorption spectrum of N,S-CDs in the presence of Cr^6+^ has a new absorption peak than that of pure N,S-CDs, which hints that the fluorescence quenching of N,S-CDs by Cr^6+^ might be a static quenching process.

As shown in Fig. 8(b), the absorption peaks in the UV-vis spectra of the Cr^6+^ were at 257 nm and 351 nm, just overlaps with the excitation as well as emission wavelength in the spectra of the N,S-CDs, which matches with the criteria of Inner Filter Effect (IFE), when there is a good spectral overlap between the absorption spectrum of the quencher and emission or excitation spectra of the same fluorophore.^41^ In order to further explore the quenching mechanism, the fluorescence lifetime of the N,S-CDs was determined (Fig. 8(c)). The results revealed that the decay curves of the prepared N,S-CDs consisted of three lifetime components and no noticeable change was observed in the average lifetime of the N,S-CDs (9.90 ns) and the N,S-CDs in the presence of Cr^6+^ (10.43 ns) (the average lifetime analysis was obtained from tri-exponential fitting), which showed a static quenching mechanism.

In order to clarify which functional groups on N,S-CDs interact with Cr^6+^, the FT-IR spectra of N,S-CDs in the absence and presence of Cr^6+^ were tested. It can be seen from Fig. 8(d) that, the addition of Cr^6+^ weakened the peak of C=O bond, N–H and O–H bond, and strengthened the peak of –O– bond. It is proved that the carboxyl or amide groups on the surface of the N,S-CDs chelate with Cr^6+^ ions, thereby strong thermodynamic combinations occur between Cr^6+^ ions and N,S-CDs.

**Fig. 9:**
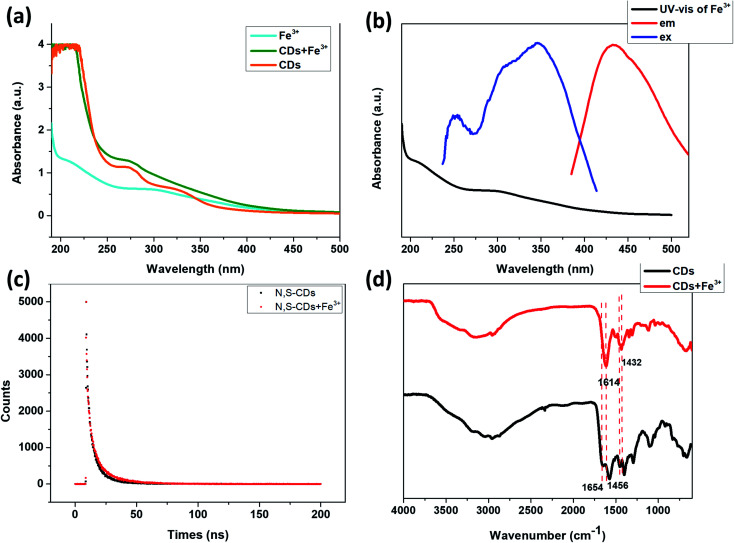
(a) UV-vis absorption spectra of Fe^3+^, N,S-CDs in the absence and presence of Fe^3+^. (b) UV-vis absorption spectra of Fe^3+^ and fluorescence excitation and emission spectra of N,S-CDs. (c) Lifetime test of N,S-CDs in the absence and presence of Fe^3+^ (excitation wavelength is at 365 nm, emission wavelength is at 434 nm). (d) FTIR spectra of N,S-CDs in the absence and presence of Fe^3+^.

The UV-vis absorption spectra of the N,S-CDs in the absence and presence of Fe^3+^ and the UV-vis absorption spectrum of Fe^3+^ (Fig. 9(a)) showed that the presence of Fe^3+^ did not significantly change the UV-vis absorption of the N,S-CDs. It can be seen from Fig. 9(b) that, the UV-vis absorption of Fe^3+^ is very weak, so the shielding effect of Fe^3+^ to the excitation light of N,S-CDs and the absorbing of the emission light of N,S-CDs are both weak, so Inner Filter Effect (IFE) could not play a dominant role in this situation.^41^ Furthermore, by comparing the fluorescence lifetime of N,S-CDs in the absence and presence of Fe^3+^ (Fig. 8(c) and 9(c)), it could be found that the fluorescence lifetime of N,S-CDs has changed significantly after adding Fe^3+^ (the average lifetime analysis was obtained from tri-exponential fitting). Therefore, the quenching of N,S-CDs by Fe^3+^ could be judged as a dynamic quenching process. Considering the Cr^6+^ cause more powerful static quenching for the N,S-CDs, while the Fe^3+^ cause weaker dynamic quenching, it was reasonable that the responsiveness of the N,S-CDs to Fe^3+^ is weaker than that to Cr^6+^.

In order to clarify which functional groups on the surface of N,S-CDs interact with Fe^3+^, the FT-IR spectra of N,S-CDs with and without Fe^3+^ were tested (Fig. 9(d)). It was found that the addition of Fe^3+^ resulted in a red-shift of the peak of the C=O bond in the FT-IR spectra of N,S-CDs, indicating that the quenching of N,S-CDs by Fe^3+^ is ascribed to the binding of Fe^3+^ to the carboxyl groups on the surface of N,S-CDs, resulting in a strong electrostatic interaction. The effect of pH on the fluorescence quenching of N,S-CDs by Fe^3+^ is determined. When the pH of the solution is 3, the fluorescence of N,S-CDs could be quenched by Fe^3+^, while when the pH of the solution is changed to 2, the degree of fluorescence quenching of N,S-CDs by Fe^3+^ is clearly weakened. This result further confirms that the quenching of N,S-CDs by Fe^3+^ is caused by the electrostatic binding of Fe^3+^ with carboxyl groups on the surface of the N,S-CDs. Under acidic conditions, carboxyl groups protonated, thus reducing the binding degree with Fe^3+^ ions.

From the above experimental investigation and theoretical mechanism exploration, it can be known that the fluorescence of N,S-CDs can be quenched by Cr^6+^ or Fe^3+^ with different quenching mechanisms. The weakening degree of the fluorescence intensity of the N,S-CDs as a function of the Cr^6+^ or Fe^3+^ concentration show a good linear relationship in a certain range, which reveals the possibility of N,S-CDs in detecting the concentration of Cr^6+^ or Fe^3+^.

### 3.7 Detection scheme when chromium and iron coexist

**Scheme 1:**
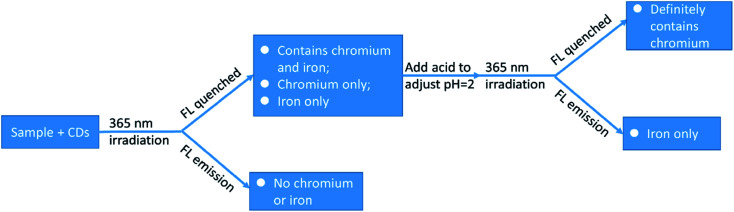
Detecting chromium or iron respectively when chromium and iron coexist.

When both are together, the iron ions interfere with the detection of chromium ions. It was found that, when the pH of the solution is 6, Fe^3+^ can make the fluorescence of carbon dots quenched, while when the pH = 2, Fe^3+^ can not quench the fluorescence of carbon dots. Hinted by this phenomenon, we designed a scheme to detect chromium ions and iron ions respectively when they are both present in the solution (Scheme 1).

### 3.8 Performance of N,S-CDs-based fluorescent hydrogel

**Fig. 10:**
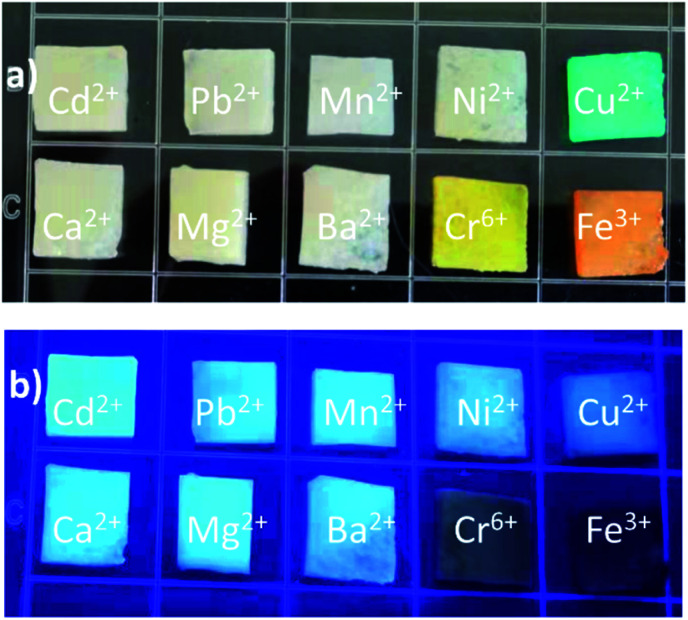
Pictures of CDs-hydrogel after being immersed into solutions containing different metal ion (a) under daylight, (b) under UV light (365 nm) (different metal ion: Cd^2+^, Pb^2+^, Mn^2+^, Ni^2+^, Cu^2+^, Ca^2+^, Mg^2+^, Ba^2+^, Cr^6+^, Fe^3+^).

**Fig. 11:**
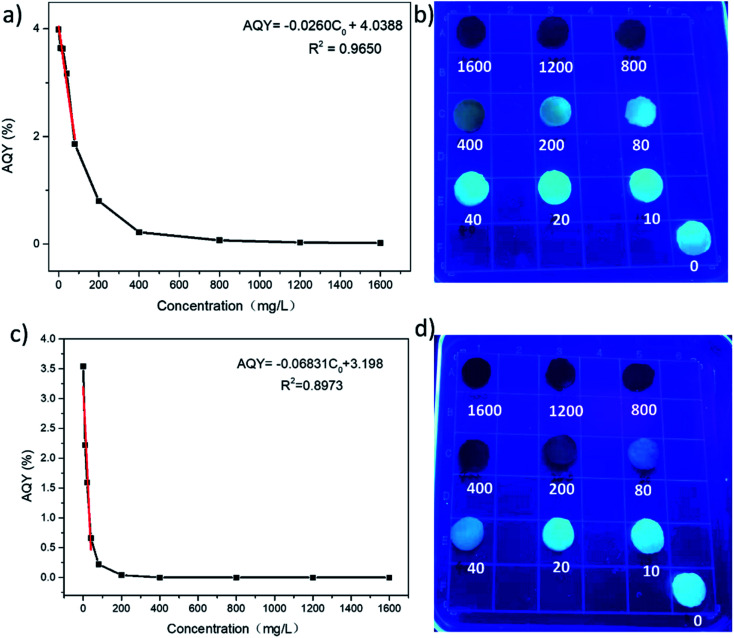
(a) The gradient fluorescence quenching of the prepared CDs-hydrogel with Cr^6+^ ion concentration. (b) Picture of CDs-hydrogel after being immersed into solutions containing different concentrations of Cr^6+^ under UV light (concentration of Cr^6+^: 1600, 1200, 800, 400, 200, 80, 40, 20, 10 mg L^−1^). (c) The gradient fluorescence quenching of the prepared CDs-hydrogel with Fe^3+^ ion concentration. (d) Picture of CDs-hydrogel after being immersed into the solutions containing different concentrations of Fe^3+^ under UV light (concentration of Fe^3+^: 1600, 1200, 800, 400, 200, 80, 40, 20, 10 mg L^−1^).

The N,S-CDs obtained by the above approach with good fluorescent properties were loaded into a hydrogel to prepare bio-friendly fluorescent hydrogel. The CDs-hydrogel samples were put into solutions containing different metal ions, and it could be found that Cr^6+^ and Fe^3+^ quenched the fluorescence of the CDs-hydrogel at low concentrations (Fig. 10). The fluorescence intensity of the CDs-hydrogel was gradually weakened with the increase of concentration of Cr^6+^ or Fe^3+^ (Fig. 11). The relative quantum yield of the CDs-hydrogel and the concentration of Cr^6+^/Fe^3+^ in the solution showed a linear relationship in a certain concentration range.

## 4. Conclusions

In this study, bio-friendly nitrogen and sulfur doped fluorescent carbon quantum dots were obtained by hydrothermal reaction using wool keratin as precursor. The effects of different reaction conditions on the relative fluorescence intensity of the prepared N,S-CDs were investigated, and the suitable reaction temperature and time were determined to be 200 °C and 10 h. The prepared N,S-CDs emit blue fluorescence under 365 nm UV irradiation, the emission wavelength depends on excitation wavelength and has wide wavelength range of light absorption. The surface of the prepared N,S-CDs is rich in functional groups such as amide, carboxyl and amino groups, so it can chelate or electrostatically interact with specific metal ion.

By determining the effect of diverse metal ions on the fluorescence properties of the N,S-CDs solution, it was found that only Cr^6+^ or Fe^3+^ caused fluorescence quenching of the N,S-CDs, and the weakening degree of the fluorescence intensity of the N,S-CDs as a function of the Cr^6+^ or Fe^3+^ concentration show a good linear relationship, thus the N,S-CDs might be used for the specific selective detection of these two metal ions. The prepared carbon dots are more sensitive to chromium ions, so the prepared carbon dots mainly used for the detection of chromium ions in chromium-rich electroplating wastewater, which will be interfered if iron ions are present, but the interference of iron can be suppressed by adding acid.

The quenching mechanism of the fluorescence of N,S-CDs by Cr^6+^ and Fe^3+^ was analyzed, and it was found that Cr^6+^ could chelate with carbonyl and carboxyl groups on the surface of N,S-CDs, which is a thermodynamic combination manner and stronger; while Fe^3+^ interacts with negatively charged groups such as carboxyl groups on the surface of N,S-CDs, which induces a dynamic quenching process.

The surface of N,S-CDs prepared in this paper is rich in functional groups, which can rapidly interact with specific metal ion, thus achieving instantaneous detection of a certain metal ion. Compared with those previous detection methods, this method with green and simple synthesis process, fast detection speed and easy and portable detection tools, is a good choice to replace the existing large detection means and has good application prospects.

## Conflicts of interest

The authors declare that they have no known competing financial interests or personal relationships that could have appeared to influence the work reported in this paper.

## Supplementary Material

RA-012-D2RA00529H-s001

## References

[cit1] Dutta D., Arya S., Kumar S. (2021). Chemosphere.

[cit2] Hayashi N., Matsumura D., Hoshina H., Ueki Y., Tsuji T., Chen J., Seko N. (2021). Sep. Purif. Technol..

[cit3] Lv Z. Q., Dong F., Zhou Z. A., Jin G. F., Sun S. H., Fu W. T. (2014). J. Alloys Compd..

[cit4] Dixit S., Yadav A., Dwivedi P. D., Das M. (2015). J. Cleaner Prod..

[cit5] Wei G., Qu J., Yu Z., Li Y., Guo Q., Qi T. (2015). Dyes Pigm..

[cit6] Ye N., Wang G., Tseng C. (2005). J. Integr. Plant Biol..

[cit7] Liu D., Liu X., Chen Z., Xu H., Ding X. (2010). Commun. Soil Sci. Plant Anal..

[cit8] Vymazal J., Březinová T. (2016). Chem. Eng. J..

[cit9] Chamjangali M. A., Goudarzi N., Mirheidari M., Bahramian B. (2011). J. Hazard. Mater..

[cit10] Anthemidis A. N., Zachariadis G. A., Kougoulis J. S., Stratis J. A. (2002). Talanta.

[cit11] Zhang J., Cheng F., Li J., Zhu J. J., Lu Y. (2016). Nano Today.

[cit12] Lim S. Y., Shen W., Gao Z. (2014). Chem. Soc. Rev..

[cit13] Wang R., Lu K., Tang Z., Xu Y. (2017). J. Mater. Chem. A.

[cit14] Baker S. N., Baker G. A. (2010). Angew. Chem., Int. Ed..

[cit15] Khayal A., Dawane V., Amin M. A., Tirth V., Yadav V. K., Algahtani A., Khan S. H., Islam S., Yadav K. K., Jeon B. (2021). Polymers.

[cit16] Zhu S., Meng Q., Wang L., Zhang J., Song Y., Jin H., Zhang K., Sun H., Wang H., Yang B. (2013). Angew. Chem., Int. Ed..

[cit17] Yang Z., Wang M., Yong A. M., Wong S. Y., Zhang X., Tan H., Chang A. Y., Li X., Wang J. (2011). Chem. Commun..

[cit18] Li L., Li L., Wang C., Liu K., Zhu R., Qiang H., Lin Y. (2015). Microchim. Acta.

[cit19] Wang Z., Qu Y., Gao X., Mu C., Bai J., Pu Q. (2014). Mater. Lett..

[cit20] Xu Y., Li H., Wang B., Liu H., Zhao L., Zhou T., Liu M., Huang N., Li Y., Ding L., Chen Y. (2018). Microchim. Acta.

[cit21] Wang Z., Xu C., Lu Y., Chen X., Yuan H., Wei G., Ye G., Chen J. (2017). Sens. Actuators, B.

[cit22] Wang D., Zhu L., Mccleese C., Burda C., Chen J., Dai L. (2016). RSC Adv..

[cit23] Wang L., Zhou H. S. (2014). Anal. Chem..

[cit24] Sahu S., Behera B., Maiti T. K., Mohapatra S. (2012). Chem. Commun..

[cit25] Wang X., Yang P., Feng Q., Meng T., Wei J., Xu C., Han J. (2019). Polymers.

[cit26] Liu S., Tian J., Wang L., Zhang Y., Qin X., Luo Y., Asiri A. M., Al-Youbi A. O., Sun X. (2012). Adv. Mater..

[cit27] Chilakamarry C. R., Mahmood S., Saffe S. N. B. M., Arifin M. A. B., Gupta A., Sikkandar M. Y., Begum S. S., Narasaiah B. (2021). 3 Biotech.

[cit28] Wang B., Yang W., McKittrick J., Meyers M. A. (2016). Prog. Mater. Sci..

[cit29] Do S., Kwon W., Rhee S. (2014). J. Mater. Chem. C.

[cit30] Dong Y., Pang H., Yang H. B., Guo C., Shao J., Chi Y., Li C. M., Yu T. (2013). Angew. Chem., Int. Ed..

[cit31] Hou J., Li J., Sun J., Ai S., Wang M. (2014). RSC Adv..

[cit32] Qu D., Zheng M., Zhang L., Zhao H., Xie Z., Jing X., Haddad R. E., Fan H., Sun Z. (2014). Sci. Rep..

[cit33] Pei S., Zhang J., Gao M., Wu D., Yang Y., Liu R. (2015). J. Colloid Interface Sci..

[cit34] Liu Z., Mo Z., Liu N., Guo R., Niu X., Zhao P., Yang X. (2020). J. Photochem. Photobiol., A.

[cit35] Iqbal A., Iqbal K., Xu L., Li B., Gong D., Liu X., Guo Y., Liu W., Qin W., Guo H. (2018). Sens. Actuators, B.

[cit36] Yao W., Hua Y., Yan Z., Wu C., Zhou F., Liu Y. (2021). RSC Adv..

[cit37] Tai D., Liu C., Liu J. (2019). Spectrosc. Lett..

[cit38] Hu G., Ge L., Li Y., Mukhtar M., Shen B., Yang D., Li J. (2020). J. Colloid Interface Sci..

[cit39] Feng S., Gao Z., Liu H., Huang J., Li X., Yang Y. (2019). Spectrochim. Acta, Part A.

[cit40] Ma Y., Chen Y., Liu J., Han Y., Ma S., Chen X. (2018). Talanta.

[cit41] Zu F., Yan F., Bai Z., Xu J., Wang Y., Huang Y., Zhou X. (2017). Microchim. Acta.

