# Supplementary material for: Green fluorescent nanomaterials for rapid detection of chromium and iron ions: wool keratin-based carbon quantum dots
Source: RSC Adv. 2022 Mar 11;12(13):8108–18. doi: 10.1039/d2ra00529h (PMC8982446; doi:10.1039/d2ra00529h)
Supplement: RA-012-D2RA00529H-s001 [file RA-012-D2RA00529H-s001.pdf]

Electronic Supplementary Material (ESI) for RSC Advances.
This journal is © The Royal Society of Chemistry 2022

Supporting information

Green fluorescent nanomaterials for rapid detection of Chromium and iron ions: wool keratin-based carbon quantum dots

Yuanyuan Song,[a] Na Qi ,[a] Kang Li,[a] Di Cheng,[a] Dong Wang[a] and Ying Li *[a]

[a] *Key Lab. of Colloid and Interface Chemistry of State Education Ministry, Shandong University, Jinan 250100, China. Email: yingli@sdu.edu.cn; Fax: +86 531 88364464; Tel: +86 531 88362078*

S2).

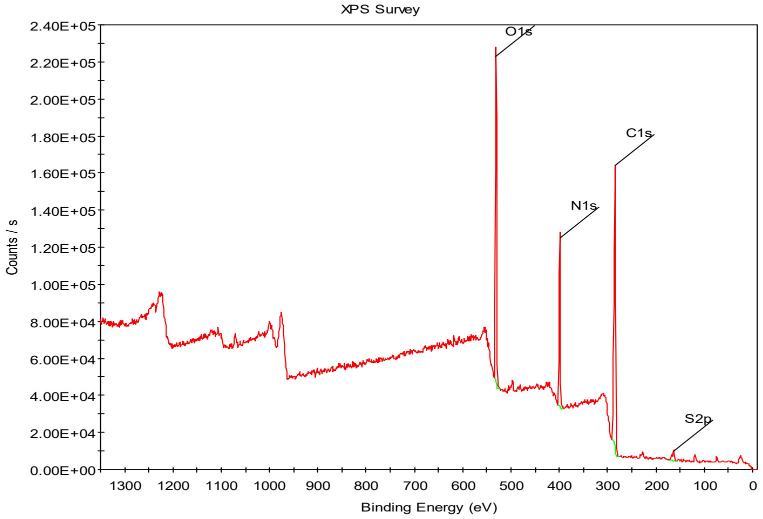

Fig. S1 XPS spectra of wool keratin.

XPS spectra of wool keratin were measured, and the peak of S element could be seen in the XPS spectra, and its content was 1.42%. (Fig. S1)

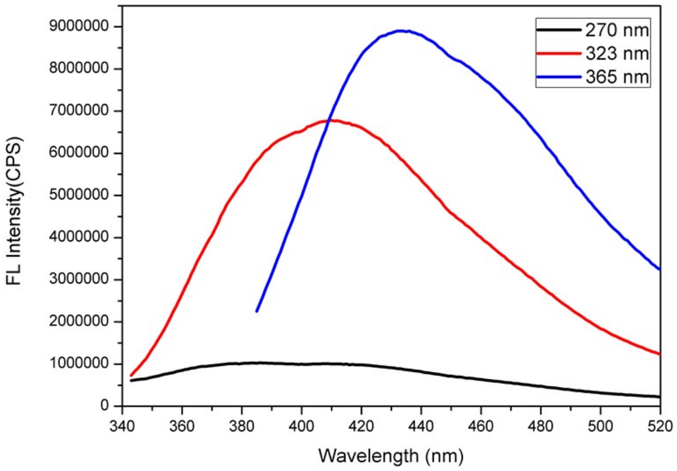

Fig. S2 Fluorescence emission spectra of prepared carbon dots under excitation at 270, 323 and 365 nm, respectively.

Fluorescence emission spectra of the prepared carbon dots have been tested when excitation wavelengths at 270 nm and 323 nm, the maximum fluorescence emission intensity is 1.03×10^{6} and 6.76×10^{6} (CPS), respectively. The intensities of fluorescence emission under excitation at 270 nm and 323 nm were weaker than that between 350-365 nm(About 8.8×10^{6}（CPS）) (Fig. S2).

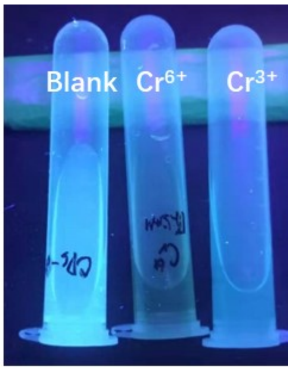

Fig. S3 Fluorescence pictures of the prepared N,S-CDs solution without, with Cr^{6+} and with Cr^{3+} under 365nm UV lamp

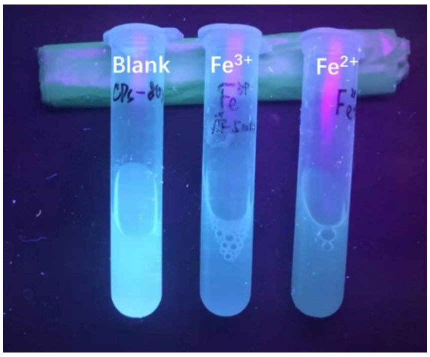

Fig. S4 Fluorescence pictures of the prepared N,S-CDs solution without, with Fe^{3+} and with Fe^{2+} under 365nm UV lamp

The effect of solutions containing the same concentrations of Cr^{6+}, Cr^{3+}, Fe^{3+}, Fe^{2+} on the fluorescence of equal amounts of the N,S-CDs solutions were determined. It was found that both Cr^{6+} and Cr^{3+} could quench the fluorescence of the prepared carbon dots, and the degree of quenching of the fluorescence of the prepared carbon dots by Cr^{3+} was weaker than that of Cr^{6+}(Fig. S3). Fe^{3+} and Fe^{2+} could both quench the fluorescence of the prepared N,S-CDs, and the degree of quenching of the fluorescence of the prepared N,S-CDs by both of them was comparable(Fig. S4).

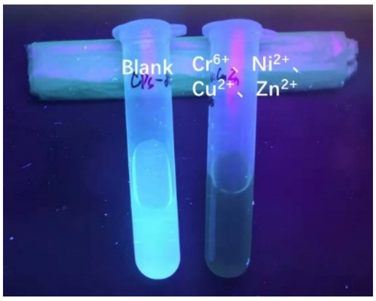

Fig. S5 Fluorescence picture of N,S-CDs solution in the absence and addition of Cr^{6+}、Ni^{2+}、Cu^{2+}、Zn^{2+} mixed solution under 365nm UV lamp

A simulating electroplating wastewater solution including Cr^{6+}, Ni^{2+}, Cu^{2+} and Zn^{2+} ions, were used to test whether the fluorescence of N,S-CDs could be quenched by chromium ions under the interference with the other types of cations. The test results showed that in the presence of Ni^{2+}, Cu^{2+} and Zn^{2+} ions, the fluorescence of the prepared carbon dots was still quenched with encountering Cr^{6+} ions(Fig. S5)

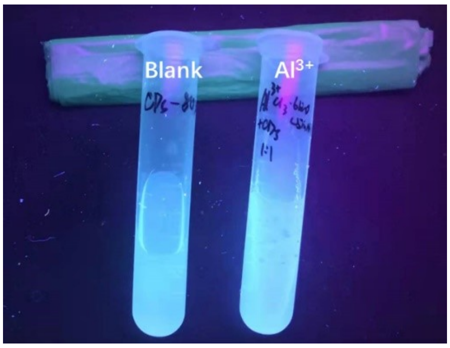

Fig. S6 Fluorescence picture of N,S-CDs solution without and with the addition of Al^{3+} solution under 365nm UV lamp

The fluorescence of N,S-CDs solution with and without Al^{3+} addition under 365nm UV lamp has been shown in the Fig. S6. When Al^{3+} ions were added to the N,S-CDs solution, the fluorescence of N,S-CDs is not quenched.
